# Hand hygiene knowledge among nurses and nursing students–a descriptive cross-sectional comparative survey using the WHO's “Hand Hygiene Knowledge Questionnaire”

**DOI:** 10.1016/j.infpip.2024.100358

**Published:** 2024-03-11

**Authors:** Per-Ola Blomgren, Christine Leo Swenne, Birgitta Lytsy, Katarina Hjelm

**Affiliations:** a Department of Public Health and Caring Sciences, Uppsala University, 751 22, Uppsala, Sweden; b Department of Medical Sciences, Unit for Clinical Microbiology and Infectious Medicine, Uppsala University Hospital, 751 23, Uppsala, Sweden

**Keywords:** Hand hygiene, Healthcare associated infections, Knowledge, Nurses, Nursing student, Questionnaire

## Abstract

**Aim:**

To determine the level of knowledge and explore the difference of hand hygiene between nursing students and nurses.

**Background:**

Annually, 3.8 million people in Europe acquire healthcare-associated infections, highlighting the importance of hand hygiene. Despite WHO's emphasis on the fact that greater hand hygiene knowledge correlates with improved hand hygiene compliance, several studies have shown knowledge gaps among nurses and nursing students regarding hand hygiene.

**Design:**

Descriptive cross-sectional comparative survey.

**Methods:**

A version of the WHO “Hand Hygiene Knowledge Questionnaire”, translated into Swedish, was used for data collection among nursing students in the first and last semester, and registered nurses from a university and associated hospital. Data were analyzed by descriptive statistics, and comparison between groups with Fisher's exact test, one-way ANOVA, and post-hoc tests (Pairwise Z-Tests, Tukey HSD).

**Results:**

The survey, conducted between December 2020 and January 2021, received responses from 201 participants, including 71 first semester students, 46 last semester students and 84 registered nurses, showing moderate (55.7% [50–74% correct answers]) to good (43.8% [75–100% correct answers]) knowledge levels. First-semester students scored lower (17.0 ± 2.1) than last-semester students (18.8 ± 1.8) and registered nurses (18.3 ± 2.1) out of 25 questions.

**Discussion:**

It is necessary for all groups to receive proper education on hand hygiene knowledge and to have an educational program that does not separate the groups but combines them with continuing education, since the students will someday be influencing future hand hygiene knowledge as a peer, together with the nurse.

## Introduction

Every year, an estimated 3.8 million people in Europe are estimated to suffer from healthcare-associated infections (HAI) [1]. The route of infection may vary, either by endogenous infection from the patient's own flora, by exogenous cross-infection through direct contact or droplets between patients or staff, or indirectly from the environment flora. Additionally, the potential for airborne transmission cannot be excluded, as pathogens may also spread through aerosols in certain conditions, further complicating the spectrum of infection risks [2]. Nursing's central aim is to promote health, but HAI pose a threat to patient well-being. The transition from health to illness or shifts in the patient's environment, caused by inadequate hand hygiene, may expose the patient to risks that affect their overall health and well-being. [3]. The cost of HAI amounts to 7 billion Euros every year due to prolonged hospital care, reoperations, and expensive medicines [4]. HAI, such as catheter-associated urinary tract infections, central-line associated bloodstream infections, pneumonia and surgical site infections can be reduced by 55–70% by adopting evidence based infection control strategies, which mainly focus on hand hygiene [5].

To strengthen patient safety and reduce the risk of harm, HAI must be prevented [6]. There are evidence-based guidelines and action bundles on how to prevent HAI. In all evidence-based guidelines, hand hygiene (HH) plays a pivotal role and is acknowledged as the foremost measure in mitigating HAI and preventing the transmission of resistant bacteria among patients [[6], [7], [8], [9]]. Practicing good HH, either by washing hands with soap and water or rubbing hands with an alcohol-based solution (hand disinfectant), is a simple and effective way to prevent the spread of microorganisms and thus HAI [6].

The Swedish Ministry of Health & Social Affairs regulates how health and medical care is to be conducted in Sweden [10]. It specifies that the care must be of good quality with a good hygienic standard. According to the National Board of Health and Welfare, the concept a good hygienic standard covers four areas related to infection prevention and control (IPC): IPC competence, premises, equipment along with organization, and planning. The concept of “IPC competence” states that all healthcare workers (HCW) must have basic knowledge of IPC as well as access to IPC expertise regarding preventive work and acute problems [11].

The WHO has produced guidelines presenting strategies on how to improve HH within healthcare settings [6]. Central to these strategies are “My Five Moments for Hand Hygiene”, which present critical moments where HCWs should perform hand hygiene: before touching a patient, before clean/aseptic procedures, after body fluid exposure/risk, after touching a patient, and after touching patient surroundings. Adhering to these moments is essential for reducing the transmission of microorganisms and improving patient safety. The strategies and implementation tools proposed by the WHO have undergone pilot testing to generate feasibility, validity, and reliability. It has been suggested that to achieve a high rate of HH adherence, HCWs need education [6]. A previous study showed a perceived absence of IPC facilitators educating HCWs and feed-back from the organization regarding compliance [12]. The individual beliefs of HCWs' regarding HH are based on their knowledge, which in turn forms their attitudes towards this practice. These attitudes guide and determine their hand hygiene behavior. Individual beliefs are influenced and learned by socialization and contact with co-workers and on an organizational level by education in institutions in the society (e.g., university, hospital) [13].

An improvement in HH correlates with knowledge about infection transmission [6]; in order to assess the level of knowledge, the WHO has designed a questionnaire that assesses knowledge on the essential aspects of HH, the Hand Hygiene Knowledge Questionnaire for Health-Care Workers [14]. Studies have shown that the knowledge level about HH among nursing students and nurses can be low to moderate in several areas such as the effectiveness of different hand hygiene agents, the impact of hand hygiene on reducing healthcare-associated infections, and specific hand hygiene practices required in different clinical scenarios [[15], [16], [17], [18], [19], [20], [21], [22], [23], [24], [25], [26], [27], [28]]. The WHO Hand Hygiene Knowledge Questionnaire has been used to assess the knowledge level of nursing students [[15], [16], [17], [18]]. These studies have graded the knowledge levels of the participants as good (scoring of 75% or above), moderate (50–74%), or poor (below 50%). Other studies have used other knowledge questionnaires [[19], [20], [21], [22], [23], [24]], with similar results. In the literature review, there are no previous studies comparing nursing students in different semesters and registered nurses (RNs). This, combined with studies showing low knowledge levels among nurses [[25], [26], [27], [28]], leads to the aims of this study. Specifically, the aim is to determine the level of knowledge and explore the difference regarding hand hygiene aspects in accordance with the WHO Hand Hygiene Knowledge Questionnaire between nursing students and RNs in Sweden. We assumed that 1st year students' knowledge would differ from last and 3rd year students; therefore, this difference will also be explored.

## Methods

### Design

A descriptive cross-sectional comparative survey, using the WHO's “Hand Hygiene Knowledge Questionnaire” [14] for data collection.

### Participants

Altogether, 487 nursing students and RNs from a single nursing program affiliated with a university hospital in Sweden were identified as eligible to participate. The eligibility criteria for inclusion in the study were: being enrolled in the nursing program at the university for students, and currently working at the university hospital for RNs. They were divided into three groups. A total survey including all nursing students in their first semester (NSS1 [n=128]), the last semester (NSS6 [n=81]), and all clinically active RNs (n=278) at 15 wards in the departments of geriatrics (n=43), medicine (n=132), and surgery (n=103) at a university hospital in Sweden.

### The questionnaire

A questionnaire was developed based on the WHO's “Hand Hygiene Knowledge Questionnaire for Health-Care Workers” [14]. It comprised 25 questions to assess knowledge of all essential aspects of HH, which focus on transmission routes for bacteria and how to prevent transmission between healthcare workers, patients, and the environment. Twelve questions were to be answered with a “yes” or “no” alternative, four with “true” or “false” statements, and nine were multiple-choice questions. Two authorized professional translators translated the original questionnaire in English through “forward-backward translation” into Swedish. The questionnaire was pilot tested on 10 nursing students and 10 RNs, resulting in minor phrasing changes.

### Data collection

Data were collected between December 2020 and January 2021 through a web-based questionnaire.

An information letter about the purpose of the study was sent out one week before the questionnaire was digitally available for all students and RNs. During the 1-month data collection, two reminders, two weeks apart, were sent out by e-mail. The nursing students received their information through their student e-mails, and the RNs received their information through secretaries and department heads who forwarded the information by e-mail. At the start of the data collection, the nursing students, in semesters 1 and 6, received oral information during one of their online lectures to encourage them to answer the questionnaire.

### Setting

The nurse training program, a three-year, two-semester-per-year curriculum leading to a bachelor's degree, integrates IPC education from the first semester. Clinical hands-on experience with patients begins in the middle of the first semester. This study was conducted during the end of their first semester for group NSS1 and during the end of the last semester for group NSS6.

The university hospital where the RNs work has an IPC unit that operates in accordance with the WHO's Multimodal Hand Hygiene Improvement Strategy [29], featuring infrastructure for improvement, training tools, evaluation, feedback mechanisms, reminders, and continual focus on patient safety. RNs have access to an annual interactive education program on hand hygiene and dress code through the education intranet software.

### Data analysis

Descriptive statistics were used, and values were given as numbers, percentages, and means (±SD). Comparisons between the groups were made with Fisher's exact test for categorical variables to measure significant differences. Post-hoc Pairwise Z-Tests were used to determine which of the groups differed from the other. Further, one-way analysis of variance (ANOVA) was made for continuous variables, followed by a post-hoc test Tukey HSD to compare groups and determine the location of dissimilarities [30]. *P*<0.05 was considered as statistically significant.

The level of knowledge, as assessed by the WHO Hand Hygiene Knowledge Questionnaire, was categorized into three groups: poor, with a score below 50%; moderate, with a score between 50 and 74%; and good, where the total correct score is 75% or higher, following the approach used in previous studies [[15], [16], [17], [18]].

Data were analyzed using the Statistical Package for the Social Sciences version 27 (SPSS Inc., Chicago, IL, U.S.A.).

### Ethical considerations

This study has been conducted in accordance with the Declaration of Helsinki [31] and Swedish law [32]. The web-based questionnaire outlined the study's purpose, voluntary participation, and confidentiality assurance. Returning the questionnaire implied consent. De-identified data were used, and no personal information was handled. Research ethics committee approval was not required as there was no processing of sensitive personal data and the study posed no physical or mental harm to the participants.

## Results

A total of 201 participants out of 487 (41%) answered the questionnaire. The participants were categorized into three groups: nursing students in their first semester (NSS1); nursing students in their last and 6th semester (NSS6); and registered nurses (RNs) working at the departments of geriatrics, medicine, and surgery. The response rate varied between the groups. NSS1 had 56% (n=71/128), NSS6 57% (n=46/81), and RNs 30% (n=84/278 [geriatrics 21%, medicine 26%, and surgery 40%]) response rate, respectively.

The majority of the responders were female (n=169, 84.1%) and the responders were aged from 19–64 years, with a mean age of 31 ± 11 years. Approximately half of the nursing students (n=63, 55.3%) had prior experience working as assistant nurses. The mean time of working as a RN was 10 ± 9.5 years. A majority of respondents (n=138, 68.7%) answered that they had received formal training in HH in the last three years, and the vast majority (n=194, 96.5%) consistently used alcohol-based hand disinfectant for HH within healthcare settings. Further information about the studied groups is provided in Table I.

**Table I:** Characteristics of the study population. First semester students (NSS1), last semester students (NSS6), and nurses (RNs)

Variable	NSS1 (n=71)	NSS6 (n=46)	RN (n=84)
Age (year) Mean± SD	24.1 ± 6.7	27.8 ± 6.4	37.8 ± 11.76
Female n (%)	56 (78.9%)	40 (87%)	73 (86.9%)
Male n (%)	15 (21.1%)	6 (13%)	11 (13.1%)
Experience of work as an assistant nurse, n (%)	29 (40.8%)	34 (73.9%)	
Years of working experience as a nurse, mean ± SD			10.4 ± 9.5
Formal training in hand hygiene in the last three years, n (%)	48 (67.6%)	39 (84.7%)	51 (60.7%)
Routinely used an alcohol-based hand disinfectant for hand hygiene within healthcare settings, n (%)	64 (90.1%)	46 (100%)	84 (100%)

### Which questions show the highest and lowest levels of knowledge among the survey groups?

In total, out of 25 questions, NSS1 had good knowledge (>75% correct answers) on 11 questions, NSS6 on 18 questions, and the RNs on 17 questions (Table II). All aspects, distributions, and differences can be seen in Table IV, Table V.

**Table II:** Number of questions from the 25 questions in the WHO Hand Hygiene Knowledge Questionnaire, with the knowledge scoring level divided by the first semester students (NSS1), last semester students (NSS6), and nurses (RN)

	NSS1	NSS6	RN
Good knowledge (>75% correct)	11	18	17
Moderate knowledge (50–74% correct)	8	2	2
Poor knowledge (<50% correct)	6	5	6

The knowledge level was considered good in 11 questions among all the groups. Regarding microorganisms (entitled ‘germs’ in the questionnaire) (Table IV), the participants had good knowledge on transmission routes for microorganisms and on how to prevent the transmission of microorganisms to the patient by using hand disinfectant before touching a patient and before a clean/aseptic procedure. They also had good knowledge on how to prevent transmission of microorganisms to the HCW by using hand disinfectant after touching a patient, after risk of contact with body fluids, and after exposure to the patient's immediate surroundings. The groups excelled with high knowledge on importance of avoiding jewellery or artificial fingernails and knowing that damaged skin increases the likelihood of colonization of harmful microorganisms on the hands.

In evaluating the appropriate hand hygiene method required in specific situations (Table V), the knowledge was good regarding the use of hand disinfectant before giving an injection and to use hand disinfectant after removing the examination gloves.

NSS6 and the RNs showed good knowledge for six additional questions, mainly regarding methods for correct HH (Table V). They knew that using hand disinfectant does not cause skin dryness more than handwashing and that the minimal time for alcohol-based hand disinfectant to kill most microorganisms is 20 seconds. In addition, they knew that hand disinfectant is the hand hygiene method required after making a patient's bed, before palpation of the abdomen and that handwashing should be used after visible exposure to blood. Considering microorganisms (Table IV), they knew that they did not need to avoid regular use of hand cream to prevent colonization of microorganisms.

The NSS6 group had one question where they had a good knowledge compared to both the NSS1 and the RN groups and that was considering washing hands after emptying a bedpan (Table V).

NSS1 and RNs had six questions with a knowledge score considered poor compared to NSS6 who had five (Table II). All groups had poor knowledge that microorganisms present on or within the patient were the most frequent source of microorganisms responsible for HAI. Additionally, they lacked understanding of hand hygiene actions taken after exposure to bodily fluids or the immediate surroundings of patients. Such preventive measures need to be performed before patient contact. Furthermore, performing hand hygiene actions to protect oneself as a HCW from microorganisms before a clean/aseptic procedure, does not prevent microorganism transmission that may occur after the procedure.

When it came to the hand hygiene method required in specific situations (Table V), there was also poor knowledge, among the groups, about handwashing and the use of hand disinfectant in sequence. These actions are not recommended to be performed in sequence.

The NSS1 and RN group also claimed that alcohol-based hand disinfectant was more effective against microorganisms than handwashing.

### How does the level of knowledge differ between the registered nurses and the nursing students?

In the survey, the mean level of knowledge regarding HH across all the groups was moderate (55.7%) to good (43.8%), as detailed in Table III. The mean score (±SD) between the groups differed; specifically, the NSS1 group's score was statistically significantly lower (17.0 ± 2.1) than the NSS6 group (18.8 ± 1.8) and the RN group (18.3 ± 2.1) (*P*=0.00) (data not shown) (where 25 was the maximum score).

**Table III:** Knowledge level measured in number of participants using the “WHO questionnaire for Hand Hygiene in Health-Care Workers” divided by the first semester students (NSS1), last semester students (NSS6), and nurses (RNs)

	NSS1 (n=71)	NSS6 (n=46)	RN (n=84)	Total (n=201)
Good >75% (score >19)	18 (25.4%)	29 (63.0%)	41 (48.8%)	88 (43.8%)
Moderate 50–74% (score between 13–19)	52 (73.2%)	17 (37.0%)	43 (51.2%)	112 (55.7%)
Poor <50% (score <13)	1 (1.4%)	0	0	1 (0.5%)

The NSS6 group had the highest level of good scores, with 63% of the participants scoring 75% points or above; the NSS1 group had the lowest number of good scores, with 25.4%; and the RN group scored lower than the NSS6, with 48.8% (Table III).

Within the area of microorganisms (Table IV), there are dissimilarities in knowledge between the groups concerning the prevention of transmission of microorganisms to the patient regarding HH action before a clean/aseptic procedure. Specifically, the NSS1 group showed lower knowledge (88.6%) compared to the NSS6 and RN groups (100% vs. 100%, *P*=0.00). Regarding preventing transmission of microorganisms to the healthcare workers, the same patterns were found concerning HH action after touching a patient (91.3% compared to 100 vs. 98.8%, *P*=0.02). The NSS1 group also showed a lower knowledge within the same area about HH action before a clean/aseptic procedure compared to the NSS6 group (13.0% vs. 31.8%, *P*=0.05) but not compared to the RNs.

**Table IV:** Hand hygiene knowledge regarding microorganisms measured with the “WHO questionnaire for Hand Hygiene in Health-Care Workers.” Correct answers marked as bold and italics. Data presented as number of correct answers (n) out of the number of respondents (*N*) “n/*N* (%).” Each subscript letter (a, b) denotes a subset of group whose proportions do not differ from each other

Item	NSS1^1^ n/*N* (%)	NSS6^2^ n/*N* (%)	RN^3^ n/*N* (%)	*P*-value
***Healthcare workers' hands when not clean*** is the main route of cross-transmission of potentially harmful germs between patients in a healthcare facility.	66/71 (93.0)	42/46 (91.3)	79/84 (94.0)	0.30
***Germs already present on or within the patient*** is the most frequent source of germs responsible for healthcare associated infections.	35/71 (49.3)	21/46 (45.7)	40/83 (48.2)	0.60
The following hand hygiene actions prevent the transmission of germs *to the patient*, answered with yes or no alternatives				
Before touching a patient (***Yes***)	70/71 (98.6)	45/46 (97.8)	84/84 (100)	0.34
Immediately after a risk of exposure to bodily fluids (***No***)	7/68 (10.3)	7/46 (15.2)	8/81 (9.9)	0.65
After exposure to the patient's immediate surroundings (***No***)	6/68 (8.8)	5/46 (10.9)	6/82 (7.3)	0.72
Immediately before a clean/aseptic procedure (***Yes***)	62/70 (88.6)_a_	46/46 (100)_b_	82/82 (100)_b_	0.00
The following hand hygiene actions prevent the transmission of germs *to the healthcare worker,* answered with yes or no alternatives				
After touching a patient (***Yes***)	63/69 (91.3)_a_	45/45 (100)_b_	83/84 (98.8)_b_	0.02
Immediately after a risk of exposure to bodily fluids (***Yes***)	68/69 (98.6)	44/46 (95.7)	83/83 (100)	0.11
Immediately before a clean/aseptic procedure (***No***)	9/69 (13.0)_a_	14/44 (31.8)_b_	14/82 (17.1)_a,b_	0.05
After exposure to the patient's immediate surroundings (***Yes***)	70/70 (100)	46/46 (100)	84/84 (100)	N/A
The following should be avoided to prevent the colonization of hands with harmful germs, answered with yes or no alternatives				
Wearing jewelry (***Yes***)	70/70 (100)	46/46 (100.0)	84/84 (100.0)	N/A
Damaged skin (***Yes***)	66/70 (94.3)	45/46 (97.8)	81/84 (96.4)	0.73
Artificial fingernails (***Yes***)	69/69 (100)	45/45 (100)	82/82 (100)	N/A
Regular use of hand cream (***No***)	46/68 (67.6)	38/45 (84.4)	68/84 (81.0)	0.07

NSS1^1^= Nursing students-semester 1, NSS6^2^= Nursing students-semester 6, RN^3^= Registered nurses.

Concerning methods for correct hand hygiene (Table V), the following dissimilarities were found between the groups. The RNs and NSS6 groups showed greater knowledge (90.4% vs. 90.7%) than the NSS1 (68.7%, *P*=0.00) on the question about hand disinfectant causing skin dryness. The NSS1 group answered correctly, to a higher extent (21.1%), than the NSS6 and RN groups (6.7% vs. 9.6%, *P*=0.05), on the false statement about handwashing and hand disinfectant being recommended to be performed in sequence.

**Table V:** Items regarding methods for correct hand hygiene. Correct answers marked as bold and italics. Data presented as number of correct answers (n) out of the number of respondents (*N*) “n/*N* (%).” Each subscript letter (a, b) denotes a subset of group whose proportions do not differ from each other

Item	NSS1^1^ n/*N* (%)	NSS6^2^ n/*N* (%)	RN^3^ n/*N* (%)	*P*-value
True statements about alcohol-based disinfectant and handwashing with soap and water are answered with true or false alternatives				
Hand disinfectant is more rapid for hand cleansing than handwashing (***True***)	36/68 (52.9)	28/45 (60.9)	38/83 (45.8)	0.21
Hand disinfectant causes skin dryness more than handwashing (***False***)	46/67 (68.7)_a_	39/43 (90.7)_b_	75/83 (90.4)_b_	0.00
Hand disinfectant is more effective against germs than handwashing (***True***)	38/68 (44.1)	21/45 (53.3)	44/83 (47.0)	0.65
Handwashing and hand disinfectant are recommended to be performed in sequence (***False***)	15/71 (21.1)_a_	3/45 (6.7)_b_	8/83 (9.6)_b_	0.05
The minimal time needed for alcohol-based hand disinfectant to kill most germs on your hands (3, 10, ***20***, 60 seconds)	48/71 (67.6)	35/46 (76.1)	66/84 (78.6)	0.22
Type of hand hygiene method required, answered with the alternatives: hand disinfectant, handwashing, or none				
Before palpation of the abdomen (***Hand Disinfectant***)	50/71 (70.4)_a_	44/46 (95.7)_b_	80/84 (95.2)_b_	0.00
Before giving an injection (***Hand Disinfectant***)	66/70 (94.3)_a,b_	40/46 (87.0)_a_	82/83 (98.8)_b_	0.02
After emptying a bedpan (***Handwashing***)	47/70 (67.1)	38/45 (84.4)	58/83 (69.9)	0.10
After removing examination gloves (***Hand Disinfectant***)	62/70 (88.6)_a_	45/46 (97.8)_a,b_	81/83 (97.6)_b_	0.04
After making a patient's bed (***Hand Disinfectant***)	45/69 (65.2)_a_	38/45 (84.4)_b_	65/83 (78.3)_a,b_	0.05
After visible exposure to blood (***Handwashing***)	52/71 (73.2)_a_	43/46 (93.5)_b_	69/84 (82.1)_a,b_	0.02

NSS1^1^ = Nursing students-semester 1, NSS6^2^ = Nursing students-semester 6, RN^3^ = Registered nurses.

When studying the type of hand hygiene method (hand disinfectant, handwashing, or none) required in certain situations, NSS1 had lower knowledge on statements about the required method before palpation of the abdomen (70.4%) than NSS6 and RNs (95.7% vs. 95.2%, *P*=0.00). This lower knowledge among NSS1 was also evident in comparison to NSS6 “after making a patient's bed” (65.2% vs. 84.4%, *P*=0.05) and “after visible exposure to blood” (73.2% vs. 93.5%, *P*=0.02). RNs had greater knowledge compared with NSS1 on “method after removing examination gloves” (97.6% vs. 88.6%, *P*=0.04), and to NSS6 regarding the required method “before giving an injection” (98.8% vs. 87.0%, *P*=0.02).

## Discussion

This study is unique since no previous studies have compared the knowledge level on hand hygiene among nursing students in different semesters during their pre-graduate education and post-graduation. The main findings were that there are knowledge gaps within all groups and that the NSS1 group had a lower knowledge compared to the NSS6 and RN groups. NSS6 had the highest level of knowledge on HH, as 63.0% had a good score. This might indicate a progression in IPC knowledge compared to NSS1, since 25.4% of the latter students had a good score. Still, the necessary HH knowledge to minimize the risk of HAI should have been 100% correct answers in the questionnaire [6]. For example, the fact that only 50% of the nursing students and RNs knew that microorganisms present on the skin or within the patient are the most frequent source of microorganisms responsible for HAI, as per the WHO hand hygiene guidelines, shows that there is room for improvement [6,29].

The questions with the lowest score in the groups concerned bacteria causing HAI and minimizing the risk of contaminating the patient and the HCW. Therefore, this study highlights that there are knowledge gaps among the nursing students and RNs, mainly the source of microorganisms causing HAI and how to prevent the transmission of microorganisms to patients and HCWs. Similar findings are found in previous studies showing a moderate level of knowledge and lacking in several areas, despite using different knowledge assessment instruments [[15], [16], [17],^19^,^24^].

One reason for a low knowledge score in the questionnaire could be the WHO methodology. The WHO is keen on promoting the 5 Moments for Hand Hygiene that follow their guidelines. These 5 moments include performing HH before and after touching a patient, before clean and dirty procedures, and after touching a patient's surroundings [29]. In Sweden, although the foundational principles of hand hygiene instruction and continuous education for HCWs are aligned with WHO guidelines, the specific terminology and expressions used to describe these moments may vary. This divergence in terminology between the international standards set by the WHO and the terminology used in Swedish nursing training programs and HCWs education could potentially lead to misunderstandings or misinterpretations of the questionnaire items, particularly those that directly refer to “My Five Moments for Hand Hygiene”, for example, the questions regarding HH actions preventing transmission of germs ‘to the patient.’ The question clearly states that transmission of germs to the patient is the focus and, therefore, HH actions performed after, for example, exposure to bodily fluids or being in the patient's surroundings, are not eligible since they occur after contact with the patient, and the HCW might now be contagious. Therefore, there might be a need to improve these knowledge gaps by revising the curriculums for the nursing students and training programs for the nurses or adapting to the WHO's My 5 Moments for Hand Hygiene.

In Sweden, nursing education at the university level and continuous education for HCWs are led by two distinct organizations. Studies to become a nurse at graduate and postgraduate levels are run by the universities while continuous education for HCWs, including nurses, are offered by the hospitals. This separation can present challenges, as continuous education has been identified as a critical component for maintaining and enhancing HCW competence [6,33,34]. Studies have highlighted that cooperation and developing joint training of students together with HCW may yield great improvements in individual knowledge and compliance [28,35]. Both organizations strive to minimize the risk of HAI, and the benefits of combining continuous education for nurses with nursing students might lead to developing a hygienic consensus. This enables a better patient safety climate by influencing HCW's individual beliefs and attitudes toward hand hygiene by being supported by the organization. The result will be a reduction in the risk of HAI and therefore risk of harm to patients and HCWs alike.

One limitation of this study is that it was conducted during the COVID-19 pandemic. The workload and well-being of the nurses might have affected the response rate, which was limited among the RNs (30%); it has been shown that healthcare workers' mental health and well-being have been affected of the COVID-19 pandemic [36]. This might be one reason why the nurses working at the departments of geriatrics and medicine, both of which managed COVID-19 patients, had a lower response rate compared to nurses working at the department of surgery, which did not have specified care for this patient group. However, the internal dropout rate was negligible, which speaks for a valid instrument to screen knowledge. Further, performing the study during the peak of the COVID-19 pandemic gave a unique possibility to study hand hygiene during a time with the highest demand on its performance.

Also, during the study, general information campaigns about COVID-19 and the importance of hygiene have been conducted throughout the world [37], and the impact of this has hopefully increased knowledge of hand hygiene, which might have affected the results in a positive way.

## Conclusion

This study determined the level of knowledge and explored the difference of registered nurses and nursing students using the WHO's hand hygiene knowledge questionnaire. The first semester students had a statistically significant lower score for knowledge on hand hygiene compared to the last semester students and registered nurses. Regarding knowledge, where the optimal aim is to know all aspects of hand hygiene in accordance with the WHO's guidelines, there were knowledge gaps within all groups. To address these gaps, we recommend the development of focused educational interventions that aligns to the specific needs of each group, emphasizing not only hand hygiene but also shaping positive attitudes towards these practices. Understanding that knowledge does not automatically translate into practice, future research should explore how attitudes influence hand hygiene compliance and the effectiveness of educational programs. This approach will be vital in fostering a culture of safety and compliance with hand hygiene standards, ultimately enhancing patient care.

## Author contributions

**Per-Ola Blomgren:** conceptualization, methodology, validation, formal analysis, investigation, data curation, writing – original draft, writing review & editing, visualisation; **Katarina Hjelm**: conceptualization, methodology, validation, supervision, project administration, funding acquisition; **Christine Leo Swenne**: conceptualization, methodology, validation, supervision; **Birgitta Lytsy**: conceptualization, methodology, validation, supervision.

## Funding

This research received no external financial or non-financial support.

## Conflict of interest statement

The authors declare no competing interests.
